# Microscopy of the Teeth

**Published:** 1867-12

**Authors:** S. P. Cutler

**Affiliations:** Professor of Chemistry and Microscopy in the Ohio Dental College, Cincinnati, formerly Professor of Chemistry and Natural Sciences in the Botanic Medical College, Memphis, Tenn.


### MICROSCOPY OF THE TEETH.

BY S. P. CUTLER, M. D., A. E. G., D. D. S.

Professor of Chemistry and Microscopy in the Ohio Dental College, Cincinnati, formerly Professor of Chemistry and Natural Sciences in the Botanic Medical College, Memphis, Tenn.

I have spoken of the process of exodentosis internas as a mixed process, or perhaps more properly, a physiological one; the result of wearing down of dentine, causing injury and wounding to tubuli, and consequently to nerve fibrils; this might be regarded as an accidental cause, and does not happen until the tooth begins to wear.

This wearing causes irritation of filaments, and in consequence a deposit of lime salts betwixt the pulp proper and dentine. This process could not be brought about unless there was an excess of lime salts deposited, that too, outside of the pulp. The salts could get there from no other source save that of the pulp; the pulp then would have to furnish materials for this process. How could this take place except that of secretion through, or rather excretion from the piumata dentalis, by a process of osmosis or osmotic action from the capillaries of that membrane, assorting the materials from the blood by a sort of elective affinity, a plus amount of os plasma at the ossified point, ossifying inwards and downwards so as to give sufficient depth to dentine to protect the pulp from the action of foreign bodies and thermal changes (as the dentine is a non-conductor), and from mechanical violence by pressure or springing of the bone down on to the pulp.

As the process advances, the pressure on the pulp from the hardening of the bone causes the nerve to absorb sufficiently to keep out of the way of pressure, which would otherwise take place to such an extent as to cause intense suffering which, however, is the case to a certain extent in certain cases. As has already been said, when this process is too much hurried, death of the pulp takes place from inflammation caused by pressure from without; the remedy is rest until the uneasiness subsides. Whether or not the dura-mata dentalis is all carried away by absorption by this process is not yet settled; so it is, there seems to be a bony union more or less complete. In this process the nerve filaments are intact, the ossific process following them down pressing away the pulp. This pulp membrane having openings where the filaments pass through, readily slips down from pressure as the bony matter accumulates around them. As this process is more or less a continuous one, I do not think there is any well developed dura mata dentalis covering this new formed dentine; at least, so long as the process is progressive, though the inner surface appears well defined and regular. When this process becomes permanently arrested from any cause, it is reasonable to suppose that there is a membraneous covering formed on the new formation, (*a posteriori.*)

At all events these views will answer my purpose until more reasonable ones are advanced to take their places. I do not say that these views are above criticism, or are entirely sound in their logic; but it is a new subject which has had but little light thrown upon it by any one, though it has been written about ere this. See Tomes' Dental Surgery, 1859.

The above remarks are not advanced fully as scientific certitudes, but as probable and reasonable hypothesis. The other form of ossification of the pulp cavity that has been spoken of is, I regard it, a different process; depending upon an entirely different cause, and might be regarded as a pathological one depending on a diseased condition of the tooth, that is, decay. There may be cases where other causes might produce the same condition of things; it might be the result of sympathetic irritation, jars or disease about the alveola, and perhaps other causes not thought of as yet. I have seen this process commenced before the decay had reached the nerve cavity, but sufficiently near to cause slight uneasiness at times; one instance of the latter character came under my own observation recently,—the tooth, an upper wisdom, had given continued, but slight uneasiness for some weeks, did not give pain by probing. On extracting the tooth there was found an ossified portion of one of the fangs which was rather larger at that point. The process commenced near the apex and continued up for a quarter of an inch, filling the canal almost completely, only on one side there being a small aperture past it. This ossification was circumscribed and oval in shape, perfectly smooth and hard, the balance of the pulp normal, not much signs of inflammatory action. There are other cases that have recently come under my observation where ossification had commenced at more than one point, then running together forming an irregular circumscribed region, not in some instances reaching to the pulp membrane. In other instances I have met with cases of molars where the entire pulp was ossified to the fangs, and a short distance in some instances, down them; have not yet met a case where the process had extended down in all the fangs to the apex, though extended observation may in future find this to be the case.

Further observation is needed, as I regard this as a very important point in Dental pathology.

In many instances the setting up or petrifying process commences immediately under the seat of the decay. If this can be regarded as a salutary or saving process, it is but reasonable to conclude that nature or the (vis medicatrix naturæ), would commence her reparations at the vulnerable point or point of attack first, and then extend as the necessity might demand. I would right here inquire, what about killing such nerves, or attempting to extract them. Would it not be advisable under all circumstances, to endeavor to ascertain whether any such process had commenced; if so, to try and aid her in her efforts, rather than to attempt to baffle nature in her salutary intentions.

There is much to learn concerning this important point in Dental pathology; many cases in my judgment might be successfully treated that are being lost.

[TO BE CONTINUED.]

